# Unicentric Pulmonary Castleman Disease Mimicking Lung Cancer: A Case Report

**DOI:** 10.1002/rcr2.70559

**Published:** 2026-03-26

**Authors:** Mingming Xu, Qing Zhang, Luyan Yin, Jianle Chen

**Affiliations:** ^1^ Department of Thoracic Surgery Affiliated Hospital of Nantong University Nantong China; ^2^ Department of Pathology Affiliated Hospital of Nantong University Nantong China; ^3^ Department of Radiology Affiliated Hospital of Nantong University Nantong China

**Keywords:** Castleman disease, lung nodule, segmentectomy

## Abstract

Castleman disease (CD) is a rare lymphoproliferative disorder typically involving mediastinal lymph nodes. Pulmonary involvement without mediastinal lymphadenopathy is extremely rare and often mimics lung cancer. A 76‐year‐old man presented with an incidental left upper lobe opacity that progressed over 3 years to a 29 mm heterogeneous enhancing nodule. Surgical resection revealed hyaline vascular unicentric CD with characteristic pathology. The patient remained disease‐free at 24 months. Pulmonary CD should be considered in enlarging lung nodules with enhancement and no mediastinal lymphadenopathy.

## Graphical abstract

A 76‐year‐old man presented with an enlarging lung nodule mimicking cancer over 3 years. Surgical resection revealed unicentric pulmonary Castleman disease with characteristic pathology. The patient remained disease‐free at 24 months.

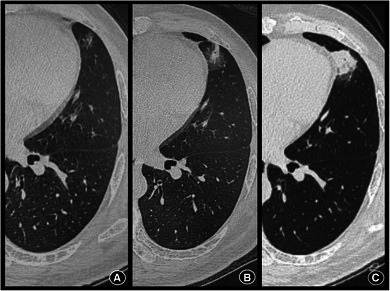

## 1 Introduction

Castleman disease (CD) is a rare lymphoproliferative disorder first described by Castleman in 1954 [1]. It is classified into unicentric CD (UCD) and multicentric CD (MCD) based on the extent of involvement [2]. UCD typically presents as localised lymphadenopathy, most commonly in the mediastinum, and surgical resection is curative [3].

Pulmonary involvement without mediastinal lymphadenopathy is extremely rare [4]. Such cases often mimic primary lung cancer on imaging, leading to preoperative misdiagnosis and unnecessary extensive resection. We report a case of UCD arising within the lung parenchyma that was initially suspected to be lung cancer and treated by pulmonary resection.

## 2 Case Report

**FIGURE 1:**
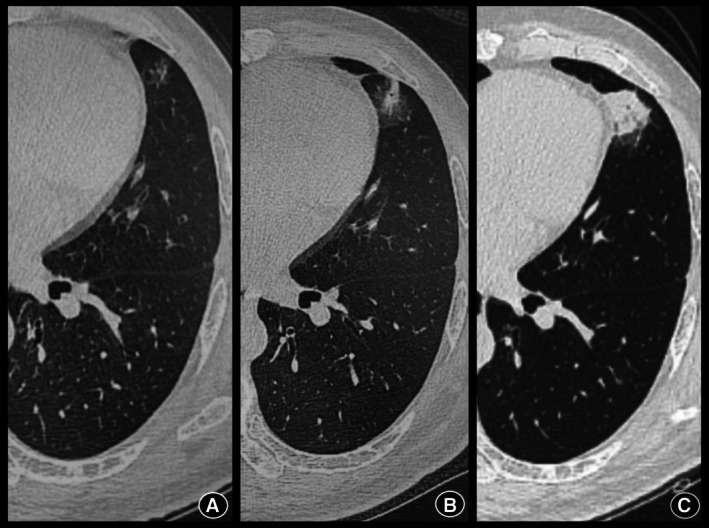
Serial chest CT images of the left upper lobe lesion. (A) September 2020: Focal ground‐glass opacity in the lingular segment. (B) February 2022: Interval enlargement to 25 mm × 21 mm with heterogeneous density. (C) June 2023: 29 mm × 26 mm mixed‐density nodule abutting the pericardium, with heterogeneous enhancement.

A 76‐year‐old man with a 50‐pack‐year smoking history and chronic obstructive pulmonary disease presented with incidental left upper lobe opacity on routine chest CT in September 2020 (Figure 1A). The lesion was initially interpreted as focal inflammation and managed conservatively. Follow‐up CT in February 2022 showed interval enlargement to 25 mm × 21 mm with heterogeneous density (Figure 1B). In June 2023, contrast‐enhanced CT revealed a 29 mm × 26 mm mixed‐density nodule abutting the pericardium, with heterogeneous enhancement (arterial phase peak 129 Hu, venous phase 58 Hu). No mediastinal or hilar lymphadenopathy was identified (Figure 1C).

The patient reported chronic cough with white sputum but no fever, night sweats, or weight loss. Physical examination was unremarkable, with no palpable lymphadenopathy. Routine laboratory tests, including complete blood count, liver and renal function, and inflammatory markers, were within normal limits. Serology for HIV was negative.

The enlarging nodule with solid components raised suspicion for primary lung adenocarcinoma. CT‐guided biopsy was considered high‐risk due to proximity to the pericardium, and the peripheral location precluded endobronchial biopsy. The patient underwent single‐port video‐assisted thoracoscopic left upper lobe lingular segmentectomy. Intraoperative findings showed mild pleural adhesions and a firm mass within the lingula. Frozen section suggested a lymphoproliferative lesion.

Macroscopically, the resected specimen contained a 4.5 cm × 2.2 cm × 2.0 cm ill‐defined, firm in consistency mass with grey‐yellow cut surface. The submitted tissue showed increased lymphoid follicles with atrophic germinal centres and expanded mantle zones. Hyalinised vessels were observed penetrating the germinal centres. No definite neurovascular invasion was identified. The lesion abutted the visceral pleural elastic layer. Airspace spread was absent, and surgical margins were negative.

**FIGURE 2:**
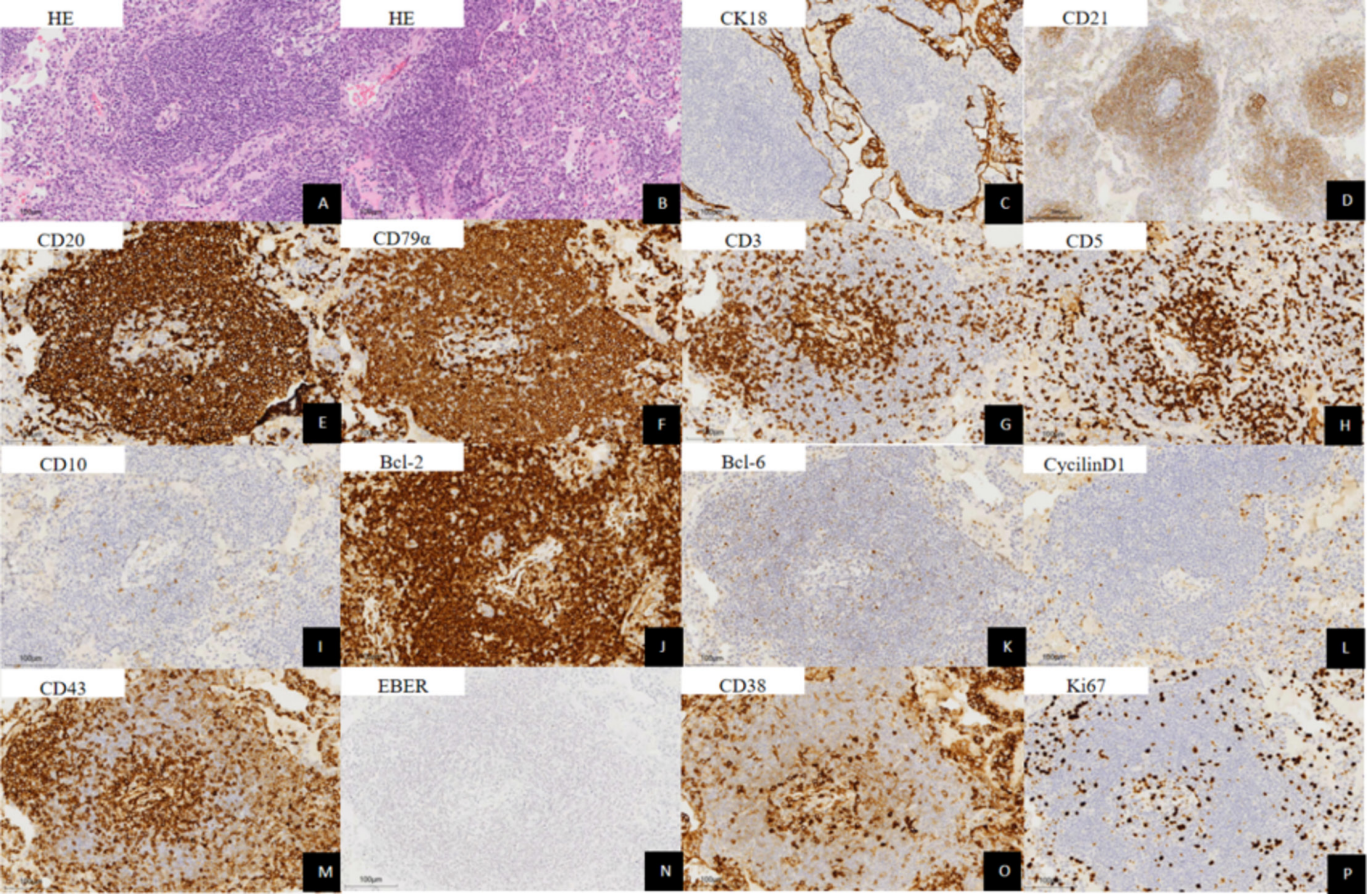
Histopathological and immunohistochemical features. (A, B) Haematoxylin and eosin staining (×400): Lymphoid follicles with atrophic germinal centres, concentric ‘onion‐skin’ mantle zones, and hyalinised vessels penetrating germinal centres (‘lollipop’ lesions). (C) CK18 (alveolar epithelial cells, positive). (D) CD21 (follicular dendritic cells, positive). (E) CD20 (B cells, positive). (F) CD79α (B cells, positive). (G) CD3 (T cells, positive). (H) CD5 (T cells, positive). (I) CD10 (negative). (J) Bcl‐2 (positive). (K) Bcl‐6 (negative). (L) Cyclin D1 (negative). (M) CD43 (positive). (N) EBER (negative). (O) CD38 (plasma cells, positive). (P) Ki‐67 (approximately 20%, positive). All immunohistochemistry: Envision method, ×400.

Immunohistochemistry (Envision method) revealed: Follicular dendritic cell marker: CD21 (+); B‐cell markers: CD20 (+), CD79α (+), Bcl‐2 (+); T‐cell markers: CD3 (+), CD5 (+), CD43 (+); Alveolar epithelium marker: CK18 (+); Plasma cell marker CD38 (+); Negative markers: CD10 (−), Bcl‐6 (−), Cyclin D1 (−), EBER (−); Proliferation index: Ki‐67 approximately 20% (Figure 2C–P). Immunoglobulin gene rearrangement: Clonal rearrangement was detected for IGH and IGL, but not for IGK. These findings confirmed the hyaline vascular subtype of CD. Surgical margins were negative (Figure 2).

The patient made an uneventful recovery and was discharged shortly after drain removal. He remained disease‐free with no evidence of recurrence on chest CT at 3, 12, and 24 months post‐surgery.

## 3 Discussion

This case highlights three important features of intrapulmonary UCD. First, the radiological evolution over 3 years—from ground‐glass opacity to mixed‐density nodule with interval growth and solidification—mimicked the natural history of indolent lung adenocarcinoma. Such a temporal course has rarely been documented for pulmonary CD. Second, the absence of mediastinal lymphadenopathy and the pure intraparenchymal location diverted the diagnosis away from CD, which is typically considered a mediastinal nodal disease. Third, despite the large size (4.5 cm) and proximity to the pericardium, complete surgical resection achieved durable remission, consistent with the favourable prognosis of UCD.

Preoperative diagnosis of pulmonary CD remains challenging. The imaging findings are non‐specific and may mimic lung cancer, tuberculosis, or organising pneumonia. In our case, the lack of calcification, cavitation, or satellite lesions argued against granulomatous infection, while the absence of ‘reverse halo’ made organising pneumonia less likely. PET‐CT or MRI, which may show hypermetabolism and prominent vessels, was not performed—a limitation acknowledged in hindsight.

Pathological examination is the gold standard. The hyaline vascular subtype, as seen here, accounts for 70%–80% of UCD and is characterised by regressed germinal centres, expanded mantle zones, and interfollicular vascular hyalinisation [3]. The EBER‐negative and polyclonal immunoglobulin gene rearrangement supported the diagnosis of unicentric CD, with no evidence of viral association or clonal malignancy.

For intrapulmonary UCD, surgery is both diagnostic and curative. Wedge resection or segmentectomy with negative margins is sufficient; mediastinal lymphadenectomy is unnecessary unless nodes are involved. Long‐term prognosis after complete excision is excellent, with a low recurrence rate.

## Author Contributions

Mingming Xu was the lead author of this case report and was responsible for acquisition of data and drafting of the work. Qing Zhang and Luyan Yin were involved in writing and reviewing the manuscript and data collection from patient records. Jianle Chen was involved in direct patient care and reviewed the manuscript. All authors approved the final version of the manuscript.

## Funding

The authors have nothing to report.

## Consent

The authors declare that written informed consent was obtained for the publication of this manuscript and accompanying images using the consent form provided by the Journal.

## Conflicts of Interest

The authors declare no conflicts of interest.

## Data Availability

The data that support the findings of this study are available from the corresponding author upon reasonable request.
